# A Rare Case of Acquired Factor VIII Deficiency in an Elderly Male With a History of Rheumatoid Arthritis

**DOI:** 10.7759/cureus.44169

**Published:** 2023-08-26

**Authors:** Shubhangi Shah, Michael Tseng, Ashimiyu Durojaiye

**Affiliations:** 1 Internal Medicine, Virginia Commonwealth University School of Medicine, Richmond, USA; 2 Hematology and Medical Oncology, Virginia Commonwealth University School of Medicine, Richmond, USA

**Keywords:** mixing study, prolonged activated partial thromboplastin time, melena, lower gastrointestinal bleeding, arteriovenous malformations, rheumatoid arthritis, clotting factor viii deficiency, auto immune, coagulation cascade, aquired hemophilia

## Abstract

Acquired hemophilia A (AHA) or factor VIII (FVIII) deficiency is caused by autoantibodies targeting FVIII in the blood coagulation pathway; it is a rare condition making it challenging to diagnose. A timely diagnosis is crucial, without which there is a risk of catastrophic bleeding. We report a case of a patient with a history of duodenal arteriovenous malformations, previously on apixaban, who presented with four days of melena. On admission he was found to have a hemoglobin of 5.7 and elevated partial thromboplastin time (PTT), promoting further workup showing FVIII levels of <1%, with a mixing study that failed to correct suggesting the presence of inhibitors against FVIII. Other characteristics of this patient’s cases included controlled rheumatoid arthritis without detectable rheumatoid factor or increased erythrocyte sedimentation rate (ESR). The patient was initially treated with prednisone and intravenous immunoglobulins, but an insufficient response prompted the initiation of recombinant factor VII, rituximab, and cyclophosphamide during hospitalization.

## Introduction

Acquired hemophilia A (AHA) or factor VIII (FVIII) deficiency is caused by autoantibodies targeting FVIII in the blood coagulation pathway [1]. It is a rare condition, with only about 1.5 million incidences every year and varies in presentation, making it challenging to diagnose [2].

FVIII is a protein in the coagulation cascade that plays a crucial role in the formation of blood clots and preventing continuous bleeding [1]. FVIII deficiency is more commonly congenital, where patients are born with an inherited mutation that leads to impaired FVIII production and requires factor replacement to prevent bleeding [3]. In its rarer form, a lack of FVIII could be due to an autoimmune process targeting the protein and depleting it [3]. An FVIII deficiency untreated could lead to catastrophic bleeding, especially in the setting of trauma [4].

We report a case of an elderly male with chronic anemia, rheumatoid arthritis, and coronary artery disease who presented with ongoing blood loss anemia likely secondary to gastrointestinal bleeding. The patient was not initially suspected to have an acquired hemophilia but was later found to have newly elevated activated partial thromboplastin time (aPTT) that prompted further workup with eventual diagnosis and management of AHA.

## Case presentation

An 84-year-old African-American man with a history of atrial fibrillation, previously on apixaban not currently on anticoagulation, rheumatoid arthritis, and coronary artery disease was admitted from in-patient rehabilitation with complaints of four days history of melena and found to have a hemoglobin of 5.7 mg/dL on presentation. The patient had three prior episodes of similar presentation within the past year with prior esophagogastroduodenoscopy that showed bleeding arterial-venous malformation (AVM) of the duodenum, which was treated with argon plasma coagulation (APC). Repeated endoscopy conducted two weeks after was unrevealing. His most recent admission a month prior was further complicated by bilateral gluteal hematoma that prompted the cessation of his apixaban. 

His diagnosis of rheumatoid arthritis was made over 20 years ago, with no known flares. His symptoms are related to only joint pain with no extra-articular manifestations.

On admission, the patient was hemodynamically stable and afebrile, with a blood pressure of 143/70 mmHg, a heart rate of 71 beats per minute, and a respiratory rate of 14 per minute. Physical exam was remarkable for subtle ecchymosis along his bilateral arms, 2+ pitting bilateral below-the-knee edema, and mild bilateral dorsal hand swelling though without warmth or significant tenderness.

Laboratory investigation revealed a prolonged aPTT of 113 seconds and a normal PT of 13.1 seconds. Testing for von Willebrand antigen and Ristocetin cofactor test were normal. A subsequent 1:1 partial thromboplastin time (PTT) mixing study failed to correct the prolonged aPTT indicating the presence of an inhibitor. FVIII activity was found to be <1% with an inhibitor titer of 204.5 Bethesda units (BU).

Additional workup revealed positive anti-nuclear antibody (ANA) of 1:640, beta-2 microglobulin of 10.08 mg/dL, and anti-cyclic citrullinated peptide of 223 units. The rheumatoid factor and lupus anticoagulant antibodies were negative. Erythrocyte sedimentation rate (ESR), C-reactive protein, C3, and C4 were all within the normal limits. All other workups, including his complete blood count (CBC) and comprehensive metabolic panel (CMP), were negative ruling out other blood coagulation disorders. He had no clinical features to suggest a malignancy. The presence of his acquired FVIII inhibitor was presumed to be related to underlying rheumatoid arthritis.

The patient was started on prednisone 100 mg daily with a six-week taper plan, along with IV immunoglobins (IVIG) 1 gram per kg for two days, with plans for further treatment on outpatient. At discharge, his PTT had been improved to 96 seconds but failed to reach normal limits. Unfortunately, the patient was re-admitted within two weeks due to the ongoing need for blood transfusion and a new hemarthrosis of the right elbow. An upper gastrointestinal endoscope on admission showed repeat bleeding of an AVM in his duodenum. Bleeding was controlled with Novo Seven (recombinant factor VII) to by-pass FVIII inhibition. He was subsequently initiated on rituximab and cyclophosphamide while hospitalized. The hospital course was complicated by worsening blood loss anemia from port sites. Despite the above therapies, he continued to have high titers of FVIII inhibitor and persistently low FVIII levels with clinical declination; the decision was made by the family and the patient to proceed with hospice care.

## Discussion

We have presented a case of an elderly gentleman presenting for blood loss acute on chronic anemia with a newly diagnosed AHA.

AHA is due to the spontaneous development of polyclonal IgG antibodies hydrolyzing FVIII into smaller fragments [4,5]. Though not well understood, the breakdown of the immune tolerance mechanism led by autoimmune disorders such as that endorsed by the patient may have proceeded this phenomenon [5]. The results of the above mechanism lead to impaired function of the intrinsic clotting cascade, manifesting as an increased risk of prolonged bleeding. Hence, one of the first signs leading to suspicion of AHA is an elevated aPTT.

Most cases of AHA are idiopathic but can be associated with malignancy, prior autoimmune conditions, infections, and certain medications. Of those with AHA and a prior autoimmune condition (11.6%), about one-third had rheumatoid arthritis, similar to our patient [5]. AHA is fairly rare with only a few cases documented in the literature from as early as 1975 and found to be associated with various conditions (Table 1). 

**Table 1:** Summary of Acquired Hemophilia A Cases in the Literature.

Table 1. Summary of Acquired Hemophilia A Cases in the Literature	
1973	77 yo M with no known medical conditions presenting with ecchymosis [6]
1998	28 yo post-partum F with no signs of disease [7]
2010	71 yo F no known autoimmune conditions, presenting with right knee swelling [7]
2013	23 yo F with no known medical conditions presenting with easy bruising and bilateral leg swelling [7]
2017	87 yo F with parvovirus B19 infection and paradoxical venous thrombosis, presenting with recurrent ecchymosis, melena, vaginal bleeding [3]
2017	59 yo F with no other known medical conditions, presenting with recurrent ecchymosis and hematomas [3]
2018	52 yo M with back myxofibroma who presented with no internal bleeding symptoms [8]
2019	68 yo F with bullous pemphigoid who presented with hemorrhagic bullae [9]
2019	35 yo post-partum F, presented with polymerous blood effusions [10]
2020	12 yo F and 18 yo M presenting with hematemesis and melena after upper abdominal surgery [11]
2021	73 yo M with chronic kidney disease and acute respiratory distress syndrome coronavirus 2 presented with large ecchymosis [12]
2023	69 yo M with no other known medical conditions and had received the second dose of the CoronaVac-SinoVac vaccine 30 days prior to consultation, presented with left leg pain from ecchymoses [13]
2023	72 yo M with chronic renal disease, presenting with subcutaneous hematomas [14]
2023	73 yo M with vitiligo on warfarin, presenting with epistaxis [14]

AHA typically presents in the elderly, but rare cases in pregnant women and pediatric populations have been seen [4]. The most common manifestation of AHA is subcutaneous bleeding, and other sites of bleeds include gastrointestinal, genitourinary, and muscle [4]. Treatment involves the management of bleeding with FVIII by-passing agents and immunosuppression via a combination of agents to address antibody formation; however, it tends to have a less favorable prognosis despite best efforts, especially in the elderly.

The development of AHA is often associated with the progression of underlying autoimmune disease [15]. Interestingly, our patient developed AHA without signs of increased disease activity of his rheumatoid arthritis. Rheumatoid factor was not detectable, with no increase in ESR, and no decreased C3 or C4 levels. He denied any joint pain or signs of an autoimmune flair.

AHA is a therapeutic emergency that usually occurs in older RAs, with 90% of cases diagnosed before a hemorrhagic syndrome [15]. Pathogenesis of the disease remained poorly understood, and the prognosis is severe. However, our patient's case is likely further exacerbated by not being on disease-modifying anti-rheumatic drugs (DMARD). 

## Conclusions

AHA is a rare condition with varying presentation, which makes diagnosis challenging. However, AHA can be life-threatening given the risk of a massive hemorrhage; thus, prompt diagnosis and treatment initiation are warranted. Our patient did not present with a soft tissue bleed similar to prior AHA cases, but he had uncontrolled gastrointestinal bleeding. Therefore, individuals with chronic anemia and unexplained internal bleeding, especially those with an autoimmune condition or malignancy, should undergo further testing to assess for acquired hemophilia. This case not only highlighted a unique presentation of an acquired FVIII deficiency but also raises awareness of the importance of early diagnosis and treatment initiation.
